# Mechanisms of Foreign Body Response Mitigation by Nitric Oxide Release

**DOI:** 10.3390/ijms231911635

**Published:** 2022-10-01

**Authors:** James B. Taylor, Maggie J. Malone-Povolny, Elizabeth P. Merricks, Lauren E. Wimsey, Daniel Soliman, Timothy C. Nichols, Shannon M. Wallet, Robert Maile, Mark H. Schoenfisch

**Affiliations:** 1 Department of Chemistry, University of North Carolina at Chapel Hill, CB3290, Chapel Hill, NC 27599, USA; 2 Department of Pathology and Laboratory Medicine, University of North Carolina at Chapel Hill, CB3290, Chapel Hill, NC 27599, USA; 3 Department of Surgery, University of North Carolina at Chapel Hill, CB3290, Chapel Hill, NC 27599, USA; 4 Division of Oral and Craniofacial Health Sciences, University of North Carolina at Chapel Hill, CB3290, Chapel Hill, NC 27599, USA; 5 Department of Microbiology and Immunology, University of North Carolina at Chapel Hill, CB3290, Chapel Hill, NC 27599, USA; 6 Curriculum of Toxicology, University of North Carolina at Chapel Hill, CB3290, Chapel Hill, NC 27599, USA; 7 Division of Pharmacoengineering and Molecular Pharmaceutics, UNC Eshelman School of Pharmacy, University of North Carolina at Chapel Hill, CB3290, Chapel Hill, NC 27599, USA

**Keywords:** foreign body response, nitric oxide, silica nanoparticles, diabetes, immune responses

## Abstract

Implantable glucose biosensors provide real-time information about blood glucose fluctuations, but their utility and accuracy are time-limited due to the foreign body response (FBR) following their insertion beneath the skin. The slow release of nitric oxide (NO), a gasotransmitter with inflammation regulatory properties, from a sensor surface has been shown to dramatically improve sensors’ analytical biocompatibility by reducing the overall FBR response. Indeed, work in a porcine model suggests that as long as the implants (sensors) continue to release NO, even at low levels, the inflammatory cell infiltration and resulting collagen density are lessened. While these studies strongly support the benefits of NO release in mitigating the FBR, the mechanisms through which exogenous NO acts on the surrounding tissue, especially under the condition of hyperglycemia, remain vague. Such knowledge would inform strategies to refine appropriate NO dosage and release kinetics for optimal therapeutic activity. In this study, we evaluated mediator, immune cell, and mRNA expression profiles in the local tissue microenvironment surrounding implanted sensors as a function of NO release, diabetes, and implantation duration. A custom porcine wound healing-centric multiplex gene array was developed for nanoString barcoding analysis. Tissues adjacent to sensors with sustained NO release abrogated the implant-induced acute and chronic FBR through modulation of the tissue-specific immune chemokine and cytokine microenvironment, resulting in decreased cellular recruitment, proliferation, and activation at both the acute (7-d) and chronic (14-d) phases of the FBR. Further, we found that sustained NO release abrogated the implant-induced acute and chronic foreign body response through modulation of mRNA encoding for key immunological signaling molecules and pathways, including STAT1 and multiple STAT1 targets including MAPK14, IRAK4, MMP2, and CXCL10. The condition of diabetes promoted a more robust FBR to the implants, which was also controlled by sustained NO release.

## 1. Introduction

Effective management of diabetes requires careful monitoring and control of blood glucose levels, to prevent hyper- and hypoglycemic episodes. While the finger-prick glucometer and corresponding treatment with insulin have led to demonstrable improvements in diabetes survivability and health outcomes, comprehensive monitoring is best achieved using implantable glucose sensors as they can provide real-time information about blood glucose fluctuations [1,2]. Unfortunately, the utility and accuracy of the glucose sensor are time-limited due to the foreign body response (FBR) in the tissue adjacent to the sensor following implantation [3].

The FBR consists of four major stages: (1) blood plasma protein adsorption to the foreign body; (2) monocyte recruitment and differentiation to macrophages; (3) macrophage activation and fusion to form giant cells; and (4) fibroblast recruitment and activation to form fibrotic tissue [4]. Stages 1 and 2 may be considered acute while stages 3 and 4 are associated with a more chronic response. The recruitment of immune cells during these different stages affects glucose sensor performance through several mechanisms including the generation of reactive oxygen and nitrogen species that can directly promote device failure, greater metabolic activity resulting in local glucose levels that are falsely lower than systemic levels, and the formation of a collagen capsule around the sensor surface such that the diffusion of glucose is slowed or even completely blocked [5,6,7,8]. Although diabetes has been shown to delay the onset of the FBR, the later stages of the FBR are accompanied by an exacerbation of these phenomena [6,9]. Several strategies have been employed in attempts to diminish the FBR and extend in vivo lifetimes of the sensor [10,11].

Nitric oxide (NO) is a diatomic gasotransmitter with many endogenous roles including angiogenesis, the regulation of inflammation, and wound healing [12,13,14,15]. These characteristics have made the active release of NO from or near the surface of the implantable sensor attractive as a means to improve the analytical biocompatibility (i.e., maintaining sensor performance by controlling the host response) [16,17,18,19,20]. Though strategies for addressing the FBR have been proposed [11,21], NO release has unique advantages. First, NO produces a uniquely localized anti-inflammatory effect due to a short in vivo half-life (5–15 s) and travel distance (~200 µm) [22,23,24]. In addition, the in situ release of NO is tunable and reliably controllable through the combination of unique NO donors [25,26] and the polymer scaffold in which they are entrapped (e.g., polyurethanes, silicones) to localize release [27,28,29,30,31,32]. To this end, we have demonstrated that the implantation of NO-releasing implants with fiber and foam topcoats greatly reduces the overall FBR response [19,20]. The first studies describing the potential utility of NO-releasing glucose sensors utilized rodent models [16,17,33,34], whereas subsequent in vivo work has leveraged porcine models due to the similarities between human and porcine tissue responses and blood flow, as well as the higher concordance between porcine studies and subsequent human translation [9,18,19,35]. Our recent work in a diabetic porcine model suggests that as long as NO release is maintained inflammatory cell infiltration and collagen density are lessened, although inflammation returns once the NO supply is exhausted [18,19,20,34]. In addition, we have demonstrated that sustained NO release promotes accurate glucose detection over time which is directly correlated with the active release of NO [19]. Though these studies strongly support the utility of NO-releasing implantable sensors for mitigating the FBR and increasing in vivo utility and duration, the exact mechanisms through which exogenous NO acts on the surrounding cells and tissue remain unclear, especially under the condition of hyperglycemia. Elucidating these mechanisms under conditions of euglycemia and hyperglycemia will inform strategies to refine appropriate NO dosage and release kinetics necessary for optimal therapeutic efficacy.

In this study, we evaluated mechanisms of action within the local tissue microenvironment using the euglycemic and hyperglycemic porcine models that were previously employed for evaluating the analytical performance of functional NO-releasing glucose sensors [19,20]. Briefly, steel wire was coated with polyurethane films containing either NO-releasing or non-releasing nanoparticles to act as a mock (i.e., non-functional) sensor replicating the geometry and NO release profile of a functional NO-releasing CGM sensor. For each experimental design, at least three NO-releasing and non-releasing mock sensors were implanted subcutaneously into the dorsum of a pig and explanted as a tissue block from the swine after either 7 or 14 d (representing acute and chronic FBR stages, respectively) of implant time along with biopsies from an area without implantation to serve as tissue-specific controls (i.e., no FBR influence). To differentiate these three sample types, they are hereafter referred to as NO-releasing, non-releasing, and control biopsies. All samples were then vertically bisected along the sensor track, with one half processed for probing the soluble mediator profile and the other half processed for immune cell and mRNA expression profiling.

## 2. Results and Discussion

### 2.1. Subcutaneously Implanted Sensors with Sustained NO Release Abrogate Sensor-Induced Acute and Chronic Foreign Body Response through Modulation of the Tissue-Specific Immune Microenvironment

Mitigation of the FBR following sensor implantation is critical to the analytical performance and use longevity of CGM devices [2]. We have previously demonstrated that sustained NO release durations of 14 and 30 d from the CGM sensor surface led to a decrease in the FBR and improved the numerical and clinical accuracies in sensor performance for >3 weeks post implantation [19]. These NO release durations were achieved using polyurethane sensor membrane coatings containing nonporous (14-d) or porous (30-d) S-nitrosothiol-functionalized silica nanoparticles [19]. In the current study, we probed the NO-mediated mechanisms of lowered FBR using non-functional or mock glucose sensors doped with porous S-nitrosothiol-functionalized silica nanoparticles (i.e., 30-d release) at the acute (7 d) and chronic (14 d) stages of the FBR. As the FBR is regulated by immune and stromal cell interactions, we first evaluated the cellular and soluble milieu of the tissues surrounding the non-releasing and NO-releasing sensors. To accomplish this, we developed a custom porcine Immunology Panel for the nanoString nCounter platform and probed the expression of mRNAs defined as being cell-type-specific to calculate cell type scores. The Immunology Panel allowed us to characterize the cellular components of the microenvironment [36]. In addition, we utilized a porcine Bioplex multiplex assay to evaluate the concentration of the following ten soluble immune mediators: GM-CSF IFNγ, IL-2, IL-4, IL-6, IL-8, IL-10, IL-12, IL-18, and TNF-α.

**Figure 1:**
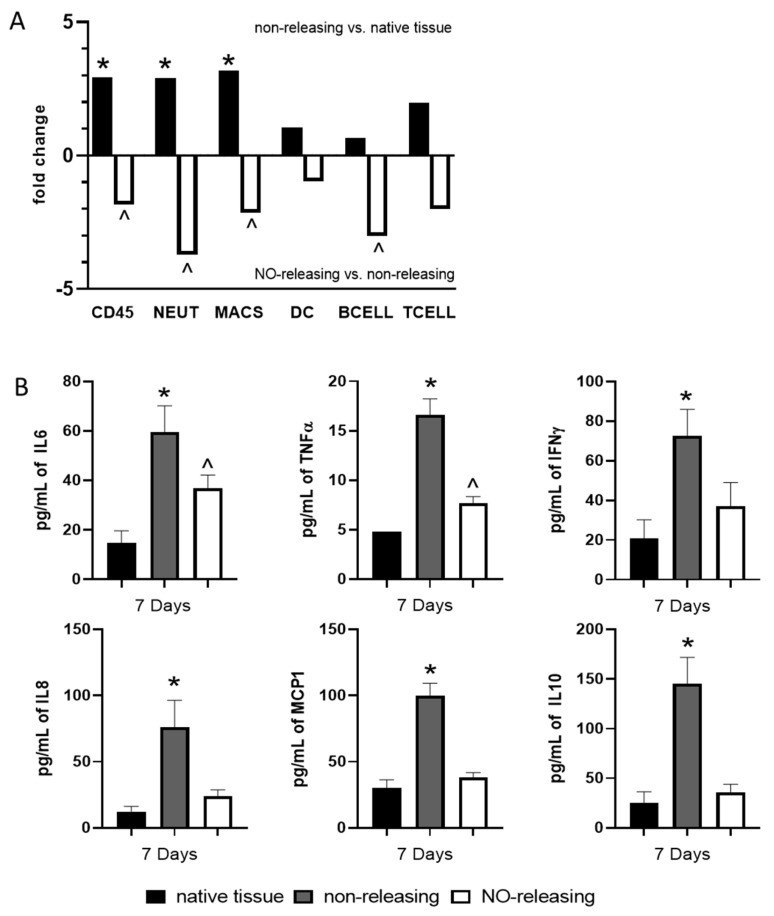
Sustained NO release abrogates the sensor-induced acute foreign body response (FBR). Native tissue or tissue surrounding mock NO-releasing or control non-releasing sensors were collected at the acute (7-d) stage of the FBR. (**A**) Expression of immune gene mRNAs canonically associated with specific immune cell populations were probed using a custom porcine nanoString panel in order to calculate cell type scores allowing us to characterize the cellular components of the microenvironment. One-way ANOVA with Bonferroni’s multiple comparisons was used to determine significance (*p* value defined as * < 0.05 non-releasing mock sensor versus native tissue; ^ < 0.05 NO-releasing versus non-releasing mock sensors; data shown are +/−SEM). (**B**) A porcine Bioplex multiplex assay was used to evaluate the concentration of ten soluble immune mediators (GM-CSF, IFNγ, IL-2, IL-4, IL-6, IL-8, IL-10, IL-12, IL-18, and TNF-α). Data were normalized to total protein determined by Braford Assay according to protocol. All cytokine concentrations are reported as the average of the three tissue samples ± the standard error of the mean. One-way ANOVA with Bonferroni’s multiple comparisons was used to determine significance (*p*-value defined as * < 0.05 non-releasing mock sensor versus native tissue; ^ < 0.05 NO-releasing versus native tissue; data shown are +/−SEM).

When evaluating the tissue microenvironment at 7 d post implantation, we observed an influx of CD45+ immune cells into the tissues surrounding non-NO-releasing sensors when compared to resting or native tissue samples, with neutrophils and macrophages the cell types most highly represented (Figure 1A). In addition, this inflammatory cell infiltration was accompanied by an induction of the pro-inflammatory cytokines IL6, TNFα, and IFNγ; the chemoattractants IL8 and MCP-1; and the immunoregulatory cytokine IL10 (Figure 1B). The soluble mediators GM-CSF, IL-2, IL4, IL-12, and IL-18 were not detected above the threshold. As expected, tissues surrounding NO-releasing sensors had significantly fewer CD45+ cells when compared to tissues surrounding non-NO-releasing sensors (Figure 1A). These data support our previous histological findings that demonstrated lower cellular density in tissues surrounding NO-releasing sensors compared to non-NO-releasing sensors [19]. The cell type scores in this study also revealed that this decreased cellular density is due to a lower frequency of neutrophils, macrophages, and β cells from tissues surrounding NO-releasing sensors (Figure 1A). This reduction in cellular infiltration was concomitant with a reduced induction of IL6, IFNγ, and IL8 and an inhibition of the induction of TNFα, MCP1, and IL10 by NO-releasing sensors (Figure 1B). Together these data indicate that the microenvironment surrounding the NO release inhibits immune cell recruitment (i.e., leading to fewer chemoattractants and CD45+ cells) and prevents a pro-inflammatory tissue-damaging response (inhibition of TNFα) within the microenvironment, resulting in a decreased acute FBR.

**Figure 2:**
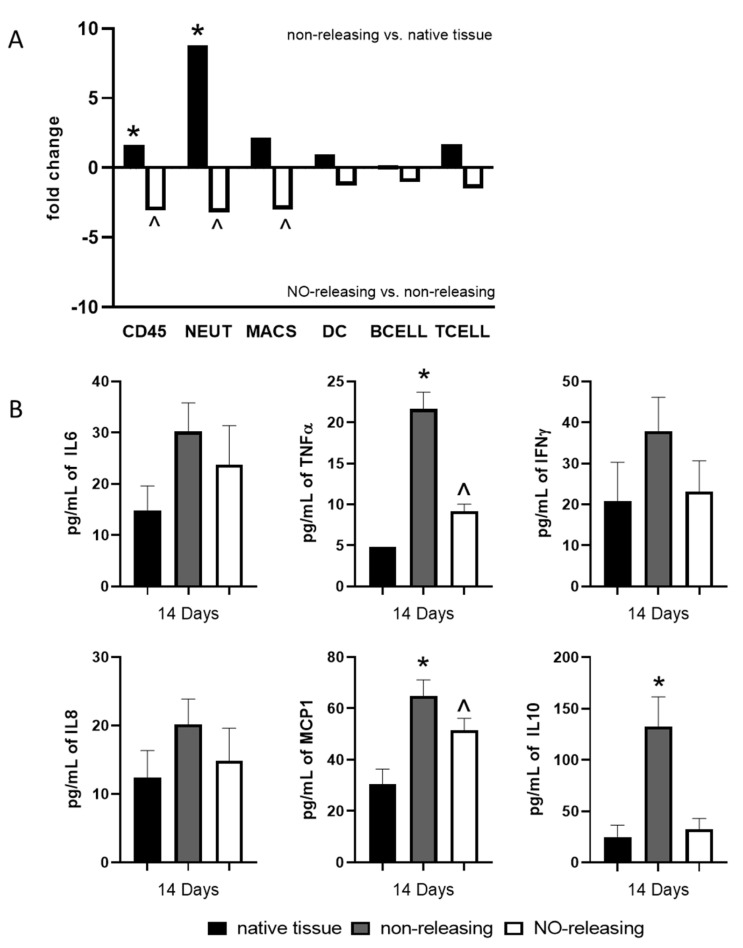
Sustained NO release abrogates the sensor-induced chronic foreign body response (FBR). Native tissue or tissue surrounding mock NO-releasing or control non-releasing sensors were collected at the chronic (14-d) stage of the FBR. (**A**) Expression of immune gene mRNAs canonically associated with specific immune cell populations were probed using a custom porcine nanoString panel in order to calculate cell type scores allowing us to characterize the cellular components of the microenvironment. One-way ANOVA with Bonferroni’s multiple comparisons was used to determine significance (*p*-value defined as * < 0.05 non-releasing mock sensor versus native tissue; ^ < 0.05 NO-releasing versus non-releasing mock sensors). (**B**) A porcine Bioplex multiplex assay was used to evaluate the concentration of ten soluble immune mediators (GM-CSF IFNγ, IL-2, IL-4, IL-6, IL-8, IL-10, IL-12, IL-18, and TNF-α). Data were normalized to total protein determined by Braford Assay according to protocol. All cytokine concentrations are reported as the average of the three tissue samples ± the standard error of the mean. One-way ANOVA with Bonferroni’s multiple comparisons was used to determine significance (*p*-value defined as * < 0.05 non-releasing mock sensor versus native tissue; ^ < 0.05 NO-releasing versus native tissue).

At 14 d post implantation, the FBR consisted of elevated levels of TNFα, MCP1, and IL10 relative to the native tissue, while the expression of IL6, IL8, and IFNγ was similar (Figure 2A). This soluble mediator milieu was concomitant with an influx of CD45+ cells (Figure 2B) from neutrophils accumulation, although a statistically significant influx of macrophages was noted as well (Figure 2B). Similar to that observed at 7 d, the NO release suppressed the local induction of TNFα and inhibited the induction of IL10 but had no effect on the MCP1 expression within the local tissues (Figure 2A). IL6, IL8, and MCP1 are potent chemoattractants for neutrophils and macrophages whereby IL6 and IL10 promote the proliferation and expansion of B cells [37]. In addition, IL6, IFNγ, and TNFα are key cytokines that induce the activation of both neutrophils and macrophages as well as the expression of often-pathogenic IgG antibodies from B cells [38]. Again, these data suggest that sustained NO release continues to modulate the tissue-specific immune microenvironment surrounding the sensor through the suppression of chemokine and cytokine expression, lessening cellular recruitment, proliferation, and activation, and thus is effective at controlling both the acute (7-d; Figure 1) and chronic (14-d; Figure 2) phases of the FBR.

### 2.2. Subcutaneously Implanted Sensors with Sustained NO Release Abrogate the Sensor-Induced Acute and Chronic Foreign Body Response through Modulation of mRNA Encoding for Key Immunological Signaling Molecules and Pathways

**Figure 3:**
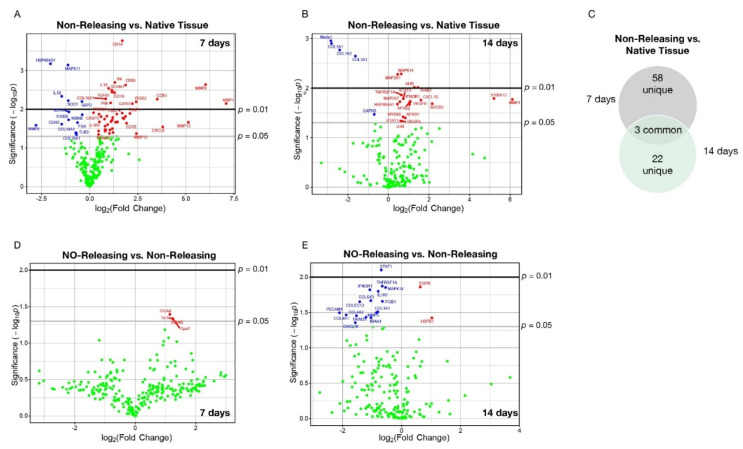
Sustained NO release abrogates the acute and chronic foreign body response (FBR) through modulation of mRNA encoding for key immunological signaling molecules. Native tissue or tissue surrounding mock NO-releasing or control non-releasing sensors were collected at the acute (7-d) and chronic (14-d) stages of the FBR. (**A**,**B**,**D**,**E**). Expression of immune gene mRNAs were probed using a custom porcine nanoString panel (254 immune-related genes). Data are presented as the log_2_-transformed differential fold change in immune gene expression, as labeled, with associated *p*-value significance (shown as −log10(*p*) and calculated using Welch’s *t*-test) after data normalization to housekeeping and internal control genes by nanoString nSolver v4.0 (Seattle, WA, USA). (**C**) Venn diagram illustrating the uniqueness and overlap of immune genes between the acute and chronic phases of the FBR.

Although our targeted analysis revealed that the NO-releasing sensors modulate the cellular and soluble mediator milieu, we also took an unbiased approach leveraging a custom porcine Immunology Panel for the nanoString nCounter platform and probed for the expression of 254 immune-related mRNAs within the explanted tissues at 7 and 14 d post implantation. Ingenuity Pathway Analysis (IPA) was then utilized to identify those pathways that were most significantly impacted by the mRNA with observed changes in expression. At seven days post implantation, unbiased analysis revealed a total of 46 and 15 mRNAs that were upregulated and downregulated, respectively, for implanted non-NO-releasing sensors (Figure 3A; Table 1). IPA revealed that these mRNAs are highly represented in and/or responsible for driving several pathways that contribute to the FBR (Supplemental Figure S1). For instance, MMP1 and MMP9 were found to be the top two induced mRNAs (7.093 log_2_-fold change; *p*-value = 0.0071 and 6.02 log_2_-fold change; *p*-value = 0.0023, respectively), with HSP90AB1 identified as the most downregulated mRNA (−2.04 log_2_-fold change; *p*-value 0.0006) in tissue surrounding non-NO-releasing sensors (Figure 3A; Table 1). Indeed, MMP9 is known to regulate pathological extracellular matrix remodeling processes that involve inflammation and fibrosis [39]. Likewise, HSP90AB1 is important for the cellular response to stress and a key player in maintaining cellular homeostasis [40]. These results provide insight into the mechanisms of our previous histological observation in the tissues surrounding non-NO-releasing sensors which revealed fibrotic encapsulation and the loss of cellular homeostasis at the site of sensor implantation [19].

mRNA expression analysis using tissues from 14 d post implantation revealed local induction of a few of the same mRNAs observed at 7 d post implantation, yet several mRNAs unique to the 14-d timepoint (Figure 3C; Table 1). Specifically, 14 d following implantation of non-NO-releasing sensors, a total of 20 and 5 mRNAs were upregulated and downregulated, respectively (Figure 3B; Table 1). The most significantly induced mRNA at 14 d was MMP1 (6.10 log_2_-fold change; *p*-value = 0.0169). Suppressed mRNAs included COL1A1, COL1A3, and COL3A1 (−2.81, −2.40, −1.63 log_2_-fold change; *p*-value ≤ 0.0023). MMP-1 is responsible for breaking down collagen types I, II, and III, while the COL gene family plays a significant role in proper collagen assembly [41]. The observed mRNA expression profile suggests a loss of extracellular matrix homeostasis, again supporting and providing mechanistic insight into previously published histological observations for NO-releasing sensors [19]. This hypothesis was also reflected in the IPA which identified several pathways implicated in the FBR (Supplemental Figure S2).

Our unbiased analysis identified only 4 mRNAs with statistically significant differences in expression in the tissue adjacent to the NO-releasing sensors versus non-releasing sensors at 7 d post implantation, each expressed at greater levels (Figure 3D; Table 2). In contrast, several differentially expressed mRNAs were identified within tissues surrounding the NO-releasing implants compared to those surrounding non-releasing glucose sensors at 14 d, and the majority were expressed at lower levels (Figure 3E; Table 2). Specifically, 2 and 17 mRNAs were expressed at greater and lesser levels, respectively, in tissues surrounding NO-releasing versus non-releasing sensors (Figure 3E; Table 2), with STAT1 being the most significantly affected mRNA (−1.68 log_2_-fold change; *p*-value = 0.0079). Of note, STAT1 is a complex protein with multiple yet contrasting transcriptional functions, whereby it acts as a positive, negative, and constitutive regulator of gene expression as well as participates in crosstalk between signal transduction pathways induced by different cytokines and growth factors that regulate cell growth, cell differentiation, the immune responses, and homeostasis [42]. This attribute of STAT1 is highlighted in our data by the concomitant suppression of the mRNA expression of several STAT1 targets including MAPK14, IRAK4, MMP2, and CXCL10 (Figure 3E; Table 2) [42]. These data also suggest that at least one driving factor for NO mitigating the FBR in the chronic phase is the modulation of STAT1 expression. We are now working to validate these unbiased analyses using targeted approaches to determine cause and effect.

### 2.3. Sensor FBR in Diabetes Controlled by Sustained NO Release

**Figure 4:**
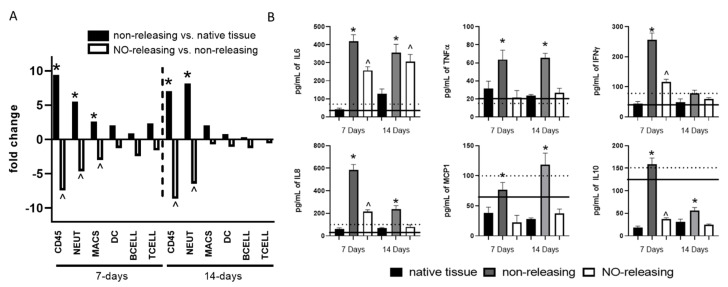
Sustained NO release prevents diabetes-induced exacerbation of the acute and chronic foreign body response (FBR). Native tissue or tissue surrounding mock NO-releasing or control non-releasing sensors from diabetic pigs were collected at the acute (7-d) and chronic (14-d) stages of the FBR. (**A**) Expression of immune gene mRNAs canonically associated with specific immune cell populations were probed using a custom porcine nanoString panel in order to calculate cell type scores allowing us to characterize the cellular components of the microenvironment. One-way ANOVA with Bonferroni’s multiple comparisons was used to determine significance (*p*-value defined as * < 0.05 non-releasing mock sensor versus native tissue; ^ < 0.05 NO-releasing versus non-releasing mock sensors). (**B**) A porcine Bioplex multiplex assay was used to evaluate the concentration of ten soluble immune mediators (GM-CSF, IFNγ, IL-2, IL-4, IL-6, IL-8, IL-10, IL-12, IL-18, and TNF-α). Dotted and solid lines represent mean cytokine levels in euglycemic animals at 7 d and 14 d after implantation, respectively. Data were normalized to total protein determined by Braford Assay according to protocol. All cytokine concentrations are reported as the average of the three tissue samples ± the standard error of the mean. One-way ANOVA with Bonferroni’s multiple comparisons was used to determine significance (*p*-value defined as * < 0.05 non-releasing mock sensor versus native tissue; ^ < 0.05 NO-releasing versus native tissue).

The condition of hyperglycemia and diabetes has profound effects on immune responses, including the FBR. As further supported by our data, the FBR to sensor implantation in a diabetic porcine model is more robust, sustained, and broader in scope. Indeed, CD45+ cells in tissues adjunct to implanted non-releasing sensors are increased by 9.5- and 7.0-fold at 7 and 14 d post implantation, respectively, when compared to levels in native tissue (Figure 4A). This significant influx of immune cells was comprised mostly of neutrophils, although the frequency of macrophages was also elevated 7 d post implantation (Figure 4A). A more robust pro-inflammatory soluble mediator milieu was also observed in the tissues surrounding non-releasing sensors in diabetic animals at both 7 and 14 d post implantation. Specifically, tissues surrounding non-releasing sensors presented with significantly greater levels of IL8, TNFα, IFNγ, IL6, MCP-1, and IL10 when compared to native tissue (Figure 4B). In addition, the levels of IL8, TNFα, IFNγ, and IL6 were greater than those observed following implantation in euglycemic animals (Figure 4B). Together, these data indicate that the FBR is more robust under the condition of hyperglycemia and may require a more aggressive approach to resolve. Thus, the efficacy of continuous NO-releasing sensors was evaluated with respect to controlling the exacerbated FBR.

When mock sensors with sustained NO release were implanted, an attenuated FBR was observed at both the acute and chronic phases. For example, at 7 d post implantation, NO-releasing sensors attenuated the induction of IL6, IFNγ, IL8, and IL10, while preventing the induction of TNFα and MCP1 (Figure 4B). Although NO-releasing sensors had no effect on IL6 expression 14 d following implantation, sustained NO release still abrogated sensor-induced expression of TNFα, IL8, MCP-1, and IL10 (Figure 4B). Similarly, NO-releasing sensors were effective at suppressing the hyperglycemia-enhanced immune infiltration, as evidenced by 7.4- and 8.6-fold fewer CD45+ cells comprised of 4.6- and 6.4-fold fewer neutrophils in the adjacent tissues relative to the non-releasing sensors at 7 and 14 d post implantation, respectively (Figure 4A). These data suggest that, even in the face of an exacerbated FBR induced by diabetes and hyperglycemia, NO-releasing sensors effectively control both the acute and chronic phases of the FBR that follows sensor implantation.

**Figure 5:**
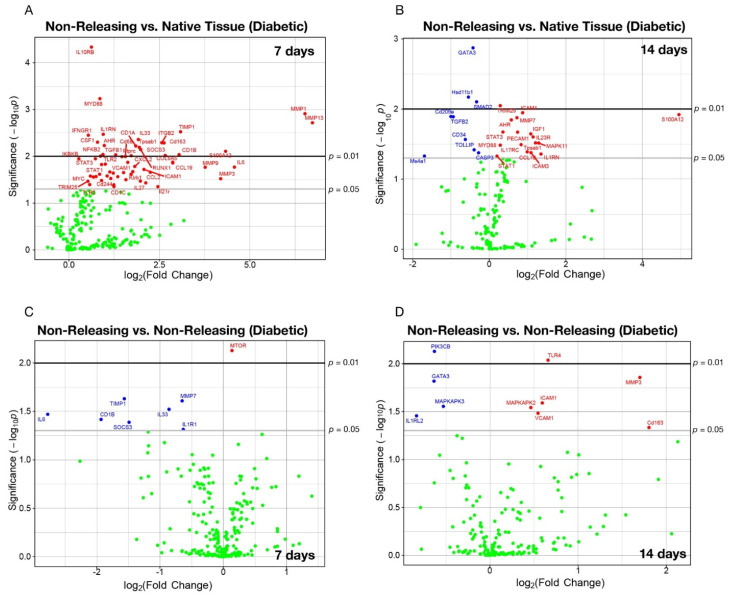
Sustained NO release abrogates the sensor-induced acute and chronic foreign body response through modulation of mRNA encoding for key immunological signaling molecules. Native tissue or tissue surrounding mock NO-releasing or control non-releasing sensors from diabetic pigs were collected at the acute (7-d) and chronic (14-d) stages of the FBR. (**A**–**D**) Expression of immune gene mRNAs were probed using a custom porcine nanoString panel (254 immune-related genes). Data are presented as the log_2_-transformed differential fold change in immune gene expression, as labeled, with associated *p*-value significance (shown as −log10(*p*) and calculated using Welch’s *t*-test) after data normalization to housekeeping and internal control genes by nanoString nSolver v4.0 (Seattle, WA, USA).

To begin to decipher mechanisms contributing to this attenuation, we again performed unbiased analysis of mRNA expression in the tissues surrounding the sensors. At 7 d post implantation, unbiased analysis revealed a total of 58 mRNAs that were differentially expressed in tissues surrounding non-releasing sensors, each found to be expressed at elevated levels when compared to native tissue from hyperglycemic animals (Figure 5A; Table 3). At 14 d post implantation, unbiased analysis disclosed 17 mRNAs that were significantly upregulated and 9 mRNAs that were significantly downregulated for the tissues surrounding non-releasing sensors in hyperglycemic animals (Figure 5B; Table 3). As above, members of the matrix metalloprotease (MMP) family proved to be the most statistically significant affected mRNAs, whereby the log_2_ fold change in expression was greater under hyperglycemic conditions than that observed in euglycemic animals (Table 1 and Table 3). Tissue MMP13 and MMP1 levels were found to have 6.73 and 6.52 log_2_-fold changes (*p*-values = 0.0019) in expression in hyperglycemic tissues surrounding non-NO-releasing sensors at 7 d implant periods (Figure 5A; Table 3). MMP3 increased expression by a 1.700 log_2_-fold change (*p*-value = 0.0139) at 14 d (Figure 5B; Table 3) compared to native tissues from hyperglycemic swine.

Similar to that observed in the euglycemic studies, the unbiased analysis identified very few mRNAs with statistically significant differences in expression surrounding NO-releasing sensors in hyperglycemic animals compared to non-releasing sensors in hyperglycemic animals at both 7 (n = 7 mRNAs) and 14 (n = 10 mRNAs) d post implantation (Figure 5C,D; Table 4). At both time points in hyperglycemic animals, nearly all of the identified mRNAs were expressed at lower levels in tissues surrounding the NO-releasing sensors relative to non-releasing sensors (Figure 5C,D; Table 4). In addition, the relative log_2_ fold changes in mRNA expression were lower than those observed in euglycemic animals (Table 2 and Table 4). For instance, the most significantly affected mRNA as measured by combined log_2_ fold change and log_10_ *p*-value was IL6 (−2.780 log_2_-fold change; *p*-value = 0.0339) and MMP3 (1.700 log_2_-fold change; *p*-value = 0.0139) at 7 and 14 d, respectively (Figure 5C,D; Table 4). Finally, IPA was utilized to delineate pathways that were driven by these mRNA profiles where, as expected, we observed that non-releasing sensors induced the upregulation of pathways that contribute to the FBR (Supplemental Figure S3A,B). The NO-releasing sensors promoted the downregulation of similar and unique pathways associated with the FBR (Supplemental Figure S4A,B).

In total, the mRNA profiles (i.e., fewer mRNAs affected and lower magnitudes of log_2_ fold change) indicate a less profound FBR for conditions of hyperglycemia and sustained NO release. Based on our data, we propose that the FBR under hyperglycemic conditions differs in both magnitude and scope, directly contributing to differences in the magnitude of log_2_ fold changes. Regardless, the results presented here demonstrate that sustained NO release to the tissue microenvironment effectively controls the aberrant FBR induced by hyperglycemia. We are now working to decipher and confirm the independent and cooperative mechanisms inferred by the mRNA expression profiles identified at both the acute and chronic stages of the FBR under the conditions of euglycemia and hyperglycemia. Such effort should complement our ongoing work focused on evaluating the sensitivity and clinical accuracy of sustained NO-releasing sensors under insulin-dependent hyperglycemia with the long-term goal of providing CGM with longer implantation lifetimes in vivo.

## 3. Materials and Methods

### 3.1. Materials

All materials were received as analytical grade and used as received unless otherwise noted. Sodium nitrite (NaNO_2_), triethylamine, cetyltrimethylammonium bromide (CTAB), concentrated hydrochloric acid (HCl), diethylenetriaminepentaacetic acid, and glutaraldehyde were purchased from Sigma-Aldrich. Ethanol (EtOH), methanol (MeOH), ammonium hydroxide (NH_4_OH, 28 wt%), anhydrous N,N-dimethylformamide (DMF), anhydrous tetrahydrofuran (THF), RNAlater RNA Isolation Solution, and T-PER protein extraction buffer were purchased from Fisher Scientific. Tetraethylorthosilicate (TEOS) and 3-mercaptopropyltrimethoxysilane (MPTMS) were obtained from Gelest and stored under nitrogen atmosphere. Polyurethanes HP93A and PC3585A were received as medical grade from Lubrizol. Nitrogen (N_2_) and nitric oxide (NO) calibration gas (25.87 ppm, balance N_2_) were purchased from Airgas National Welders. Water was purified using a Millipore water purification system to a resistivity of 18.2 MΩ cm and a total organic content of <6 ppb.

### 3.2. Particle Synthesis and Preparation of Sensors

Porous silica nanoparticles were synthesized as reported in previously published literature [19,43]. Briefly, a bolus of TEOS was injected into a solution of ethanol, water, NH_4_Oh, and CTAB. Particles were washed with ethanol, isolated with centrifugation, and post-grafted with MPTMS, thiolating the interior and exterior of the particle. These particles were then nitrosated in a solution of acidified nitrite at 0 °C. Particles were synthesized and handled in the dark to prevent premature light-activated NO release. Non-releasing particles were synthesized in the same manner, omitting the nitrosation step.

Mock glucose sensors were coated with polyurethanes containing either NO-releasing or non-releasing nanoparticles. The mock sensor was designed to replicate the geometry and NO release profile of an NO-releasing glucose biosensor, with an initial NO flux of roughly 6 pmol cm^−2^ s^−1^ that decreased to roughly 2 and 1 pmol cm^−2^ s^−1^ by day 7 and 14, respectively [19,43]. These sensors were fabricated in a biosafety cabinet by first sterilizing steel wire in 5% glutaraldehyde for 1 h and loop casting (i.e., 6.5 µL solution applied using a 2 mm steel wire loop) 10 coats of 25 mg mL^−1^ particles:25 mg mL^−1^ HP93A in 3:1 THF:DMF, with 4 min drying between each coat. A single loop cast of 3:1 PC3585A:HP93A in THF:DMF was applied, followed by a 30 min air dry. Implants were fabricated < 12 h prior to implantation and stored in centrifuge tubes contained in vacuum-sealed bags and kept at −20 °C until usage. Sensors were brought to room temperature ~2 min before implantation.

### 3.3. In Vivo Assessment of Foreign Body Response

All procedures and protocols were in accordance with institutional guidelines and approved by the Institutional Animal Care and Use Committee at the University of North Carolina in Chapel Hill. Sinclair piglets were utilized as euglycemic (n = 1) and diabetic (n = 1) porcine models [44].

Nitric oxide-releasing (n = 3) and non-releasing (n = 3) mock sensors were implanted subcutaneously into the dorsum of each swine as previously published [19]. Briefly, pigs were sedated via a Telazol injection, with long-term anesthesia maintained with propofol. An NO-releasing glucose biosensor and a control glucose biosensor of a given particle dopant were implanted in pairs along the spine. Implantation was accomplished by initially inserting an 18G catheter in the subcutis. A sensor was threaded into the cannula, and then the cannula was removed, leaving the sensor implanted percutaneously. This insertion method likely causes tissue trauma induced by the catheter. The effect of this trauma was kept consistent between insertions and minimized by choosing the smallest gauge needle into which the sensor could be threaded (18G). Sensors were secured using a suture and surgical glue at the insertion site to minimize sensor micromotion within the tissue.

Tissue samples surrounding the sensors were surgically excised as a block using a punch biopsy at 7 and 14 d after sensor implantation and processed for mRNA and protein lysates. Biopsies were also obtained on day 0 (i.e., implantation day) and day 14 from an area without implants to serve as tissue-specific controls. Samples were vertically bisected along the sensor track, with each half placed into either 0.6 mL of T-PER protein extraction buffer or 1 mL of RNAlater for protein and mRNA analysis, respectively. Samples in protein extraction buffer were frozen immediately in a −80 °C freezer. The samples in RNAlater were stored in a 4 °C fridge for < 1 w to promote full perfusion into the tissue and then stored at −20 until mRNA could be extracted.

### 3.4. Soluble Mediator Expression

Tissues were thawed and homogenized in T-PER and the homogenate was subjected to multiplex analysis using MILLIPLEX MAP Porcine Cytokine/Chemokine Magnetic Bead Panel—Immunology Multiplex Assay, according to manufacturer protocol, which probed for the expression of GM-CSF IFNγ, IL-1α, IL-1β, IL-1ra, IL-2, IL-4, IL-6, IL-8, IL-10, IL-12, IL-18, and TNF-α. Briefly, protein lysates and cytokine capture-bead cocktails were incubated for 2 h. The samples were then incubated for 1.5 h with biotin-labeled anti-cytokine and then for 30 min in a 1:12.5 dilution of streptavidin-phycoerythrin. Data were acquired on a Bioplex 200 and analyzed with Milliplex Analyst software using 5 parameter logistics and standard curves. Data were normalized to total protein determined by Braford Assay according to protocol. All cytokine concentrations are reported as the average of the three tissue samples ± the standard error of the mean. One-way ANOVA with Bonferroni’s multiple comparisons was used to determine significance (*p*-value < 0.05).

### 3.5. mRNA Expression

Tissues were thawed and homogenized utilizing Qiagen TissueLyser II according to the manufacturer’s instructions. mRNA was isolated using a Qiagen RNeasy Fibrous Mini kit, including DNase I treatment, according to protocol. mRNA was eluted with 45 μL of nuclease-free water and stored at −80 °C until nanoString could be performed. mRNA was quantified using a Qubit fluorometer and brought to a concentration of 20 ng/μL; 100 ng of mRNA was used for porcine nanoString nCounter mRNA microarray assay according to the manufacturer’s instructions. These mRNAs were hybridized to probes at 65 °C for 16 h. Hybridized probes were extended and quantified using the nCounter Prep Station and Digital Analyzer. The nCounter-generated relative fluorescent intensities were analyzed using nSolver Advanced Analysis 4.0 software according to the manufacturer’s instructions. Data from each sample were normalized using built-in positive controls to control for technique in hybridization, housekeeping mRNAs to account for varying sample degradation, and built-in negative controls to account for background signal. Samples were grouped according to their parameters, and, for each mRNA, fold changes between groups were calculated using the mean of the normalized samples. Abundance of various cell populations was also calculated using the Nanostring Cell Type Profiling Module. In brief, cell populations were quantified using marker mRNAs which are expressed stably and specifically in given cell types. A filtering of data with *p*-values less than or equal to 0.05 was performed. Differential expression volcano plots were generated using logarithmically transformed fold changes of averaged normalized counts for each group using the reference group as indicated. Differential expression was analyzed using Ingenuity Pathway Analysis (IPA) to identify pathways that were significantly represented by the differential mRNA expression.

### 3.6. Statistical Analysis

Normality of data was determined using a D’Agostino–Pearson test, whereby continuous variables were compared using Mann–Whitney or Kruskal–Wallis, with Dunn’s multiple comparison and analysis performed using GraphPad Prism v9.0. For nanoString data, a negative binomial mixture model, simplified negative binomial model, or loglinear model were used depending on each mRNA’s mean expression compared to the background threshold. Multiple testing correction was performed using the method of Benjamini–Yekutieli. Causal Network Analysis was performed using IPA.

## Figures and Tables

**Table 1:** mRNAs that were significantly upregulated and significantly downregulated within tissues surrounding non-NO-releasing sensors 7 and 14 d following implantation in euglycemic animals.

7 d Post Implantation						14 d Post Implantation		
mRNA	log_2_ Fold Change	*p*-Value	mRNA	log_2_ Fold Change	*p*-Value	mRNA	log_2_ Fold Change	*p*-Value
MMP1	7.093	0.0071	CCL2	1.029	0.0421	MMP1	6.110	0.0169
MMP9	6.020	0.0023	IL18	0.986	0.0029	S100A12	5.210	0.0162
MMP13	5.128	0.0217	CASP1	0.934	0.0249	SOCS3	2.160	0.0206
CXCL8	3.799	0.0285	Tpsab1	0.895	0.0149	CXCL10	1.600	0.0174
CCR1	3.513	0.0055	COL16A1	0.832	0.0054	IRAK3	1.450	0.0097
MMP12	2.428	0.0424	COL5A3	0.832	0.0338	AHR	1.280	0.0096
ITGB2	2.417	0.0064	PECAM1	0.807	0.0369	IFNGR1	1.080	0.0185
Cd163	2.217	0.0072	SOCS1	0.799	0.0272	VEGFB	1.040	0.0213
TIMP1	2.094	0.0128	TLR2	0.799	0.0335	NFKB2	1.030	0.0200
CD1B	1.918	0.0229	IL10RB	0.774	0.0179	NFKB1	0.831	0.0399
CD86	1.868	0.0024	MYD88	0.595	0.0155	VEGFA	0.819	0.0485
Cd68	1.864	0.0153	IL1R1	0.506	0.0211	STAT3	0.730	0.0164
Csf3r	1.774	0.0174	CASP3	0.485	0.0134	MYD88	0.727	0.0379
ICAM1	1.731	0.0168	CD40LG	0.465	0.0362	JUN	0.697	0.0470
CD14	1.696	0.0002	TRIM26	0.465	0.0446	TNFRSF1A	0.642	0.0138
Nkg7	1.692	0.0125	TRIM25	0.202	0.0123	MAPK14	0.631	0.0052
Ptprc	1.546	0.0104	IKBKB	−0.390	0.0138	STAT1	0.605	0.0460
Fcgr4	1.444	0.0212	G6PD	−0.401	0.0063	MAP3K7	0.566	0.0188
RUNX1	1.411	0.0133	FGA	−0.632	0.0220	MAP2K1	0.449	0.0053
CCR5	1.350	0.0216	COL10A1	−0.687	0.0456	HSP90AA1	0.440	0.0210
Blk	1.310	0.0020	TLR3	−0.696	0.0407	CAPN5	−0.698	0.0339
CD1A	1.293	0.0037	COL14A1	−0.722	0.0404	COL3A1	−1.630	0.0023
CCR2	1.214	0.0396	SDHA	−1.077	0.0117	COL1A2	−2.400	0.0017
IL33	1.202	0.0163	S100B	−1.098	0.0187	COL1A1	−2.810	0.0013
MMP14	1.176	0.0317	SOD1	−1.111	0.0060	Ms4a1	−2.840	0.0011
COL7A1	1.170	0.0190	MAPK11	−1.124	0.0007			
TGFB1	1.163	0.0036	IL1B	−1.454	0.0046			
VCAM1	1.151	0.0032	CDH2	−1.454	0.0242			
CCL19	1.104	0.0208	HSP90AB1	−2.036	0.0007			
Hdc	1.091	0.0068	MMP8	−2.789	0.0258			
Cd3d	1.050	0.0342						

**Table 2:** mRNAs that were expressed at significantly higher levels and significantly lower levels in tissues surrounding NO-releasing sensors compared to those surrounding non-releasing sensors at 7 and 14 d following implantation in euglycemic animals.

7 d Post Implantation			14 d Post Implantation		
mRNA	log_2_ Fold Change	*p*-Value	mRNA	log_2_ Fold Change	*p*-Value
Cpa3	2.370	0.0464	HSPB1	1.030	0.0377
ICAM5	2.370	0.0464	EGFR	0.632	0.0138
Tcl1	2.370	0.0464	MAPK14	−0.542	0.0140
ITGA4	2.210	0.0404	ITGB1	−0.650	0.0220
			TNFRSF1A	−0.655	0.0134
			STAT1	−0.689	0.0079
			IL1R1	−0.791	0.0159
			COL3A1	−0.806	0.0309
			MMP2	−0.856	0.0319
			COL6A3	−1.040	0.0215
			IRAK4	−1.040	0.0375
			IFNGR1	−1.080	0.0151
			TRIM25	−1.210	0.0369
			COLEC12	−1.420	0.0223
			COL4A2	−1.530	0.0352
			CXCL10	−1.570	0.0441
			COL4A1	−1.880	0.0342
			PECAM1	−2.110	0.0319

**Table 3:** mRNAs that were significantly upregulated and significantly downregulated within tissues surrounding non-NO-releasing sensors 7 and 14 d following implantation in hyperglycemic animals.

7 d Post Implantation						14 d Post Implantation		
mRNA	log_2_ Fold Change	*p*-Value	mRNA	log_2_ Fold Change	*p*-Value	mRNA	log_2_ Fold Change	*p*-Value
MMP13	6.730	0.0019	Cd209e	1.510	0.0223	S100A12	4.960	0.0120
MMP1	6.520	0.0012	Fcgr4	1.460	0.0102	IL1RN	1.350	0.0438
IL6	4.570	0.0170	ITGAL	1.350	0.0273	MAPK11	1.290	0.0306
S100A12	4.330	0.0078	TGFB1	1.290	0.0093	Tpsab1	1.210	0.0304
MMP3	4.200	0.0301	Cd244a	1.240	0.0416	IL23R	1.140	0.0250
MMP9	3.760	0.0171	CD1C	1.240	0.0443	ICAM3	1.090	0.0419
TIMP1	3.080	0.0030	CXCL10	1.220	0.0229	IGF1	1.080	0.0226
CD1B	3.040	0.0092	MMP2	1.160	0.0298	CCL19	0.994	0.0408
COL6A5	2.870	0.0134	IL1R1	1.130	0.0216	ICAM1	0.872	0.0113
CCL19	2.870	0.0139	Ms4a2	1.060	0.0263	IL17RC	0.832	0.0322
SOCS3	2.660	0.0096	TLR2	1.010	0.0147	PECAM1	0.736	0.0214
Cd163	2.630	0.0052	AHR	0.982	0.0059	MMP7	0.719	0.0133
ITGB2	2.570	0.0051	IL1RN	0.952	0.0034	AHR	0.573	0.0143
Il21r	2.460	0.0445	NFKB2	0.939	0.0092	STAT3	0.360	0.0213
CCL2	2.250	0.0220	STAT1	0.906	0.0150	MYD88	0.292	0.0328
IL27	2.130	0.0370	MMP7	0.903	0.0330	TRIM25	0.289	0.0090
ICAM1	2.070	0.0191	TNFRSF1A	0.874	0.0099	STAT1	0.199	0.0470
RUNX1	1.980	0.0072	MYD88	0.856	0.0006	CASP3	−0.280	0.0421
Klrb1	1.970	0.0339	TRIM8	0.849	0.0242	SMAD2	−0.332	0.0079
Tpsab1	1.950	0.0064	CSF1	0.794	0.0050	TOLLIP	−0.396	0.0382
IL33	1.920	0.0044	IL17RA	0.752	0.0271	GATA3	−0.425	0.0013
CD1A	1.850	0.0060	STAT3	0.728	0.0113	Hsd11b1	−0.544	0.0068
CXCL2	1.820	0.0165	MMP14	0.678	0.0278	CD34	−0.625	0.0272
CCR5	1.760	0.0212	IL10RB	0.619	0.0000	TGFB2	−0.941	0.0129
Ptprc	1.720	0.0097	MYC	0.593	0.0265	Cd209e	−1.000	0.0128
CD86	1.720	0.0242	TLR3	0.572	0.0404	Ms4a1	−1.700	0.0469
VCAM1	1.630	0.0135	IFNGR1	0.534	0.0036			
Ms4a4a	1.580	0.0316	TRIM25	0.526	0.0342			
Cd68	1.560	0.0101	IKBKB	0.278	0.0113			

**Table 4:** mRNAs that were expressed at significantly higher levels and significantly lower levels in tissues surrounding NO-releasing sensors compared to those surrounding non-releasing sensors at 7 and 14 d following implantation in hyperglycemic animals.

7 d Post Implantation			14 d Post Implantation		
mRNA	log_2_ Fold Change	*p*-Value	mRNA	log_2_ Fold Change	*p*-Value
MTOR	0.139	0.0074	Cd163	1.810	0.0464
MMP7	−0.652	0.0246	MMP3	1.700	0.0139
IL33	−0.856	0.0301	TLR4	0.660	0.0092
SOCS3	−1.490	0.0410	ICAM1	0.594	0.0257
TIMP1	−1.570	0.0234	VCAM1	0.544	0.0329
CD1B	−1.930	0.0383	MAPKAPK2	0.469	0.0287
IL6	−2.780	0.0339	MAPKAPK3	−0.528	0.0279
			PIK3CB	−0.628	0.0074
			GATA3	−0.633	0.0152
			IL1RL2	−0.831	0.0350

## Data Availability

Not applicable.

## Supplementary Materials

The supporting information can be downloaded at: https://www.mdpi.com/article/10.3390/ijms231911635/s1.
