# Supplementary material for: Mechanisms of Foreign Body Response Mitigation by Nitric Oxide Release
Source: Int J Mol Sci. 2022 Oct 1;23(19):11635. doi: 10.3390/ijms231911635 (PMC9569454; doi:10.3390/ijms231911635)

Supplemental Figure S1. Ingenuity Pathway Analysis (IPA) revealed pathways in which mRNA identified to be differentially expressed in tissues surrounding non-releasing sensors 7-d post implantation are highly represented. Native tissue and tissue surrounding control non-releasing sensors were collected at the acute (7-d) stage of the foreign body response (FBR). Expression of immune gene mRNAs were probed using a custom porcine nanoString panel (254 immune related genes). Ingenuity Pathways Analysis (IPA) was employed to detect and visualize the significant changes in canonical immune signaling genes based on the observed gene expression profiles (Figure 3A). Data are presented as the Bayesian z-scores with associated p-value significance. Z-scores represent the degree of perturbation of the group of pathway genes versus the controls. Positive z-scores represent activation of the pathway (pathway score is dominated by more up-regulated genes), while the negative z-score represents pathway repression (pathway score is dominated by down-regulated genes).

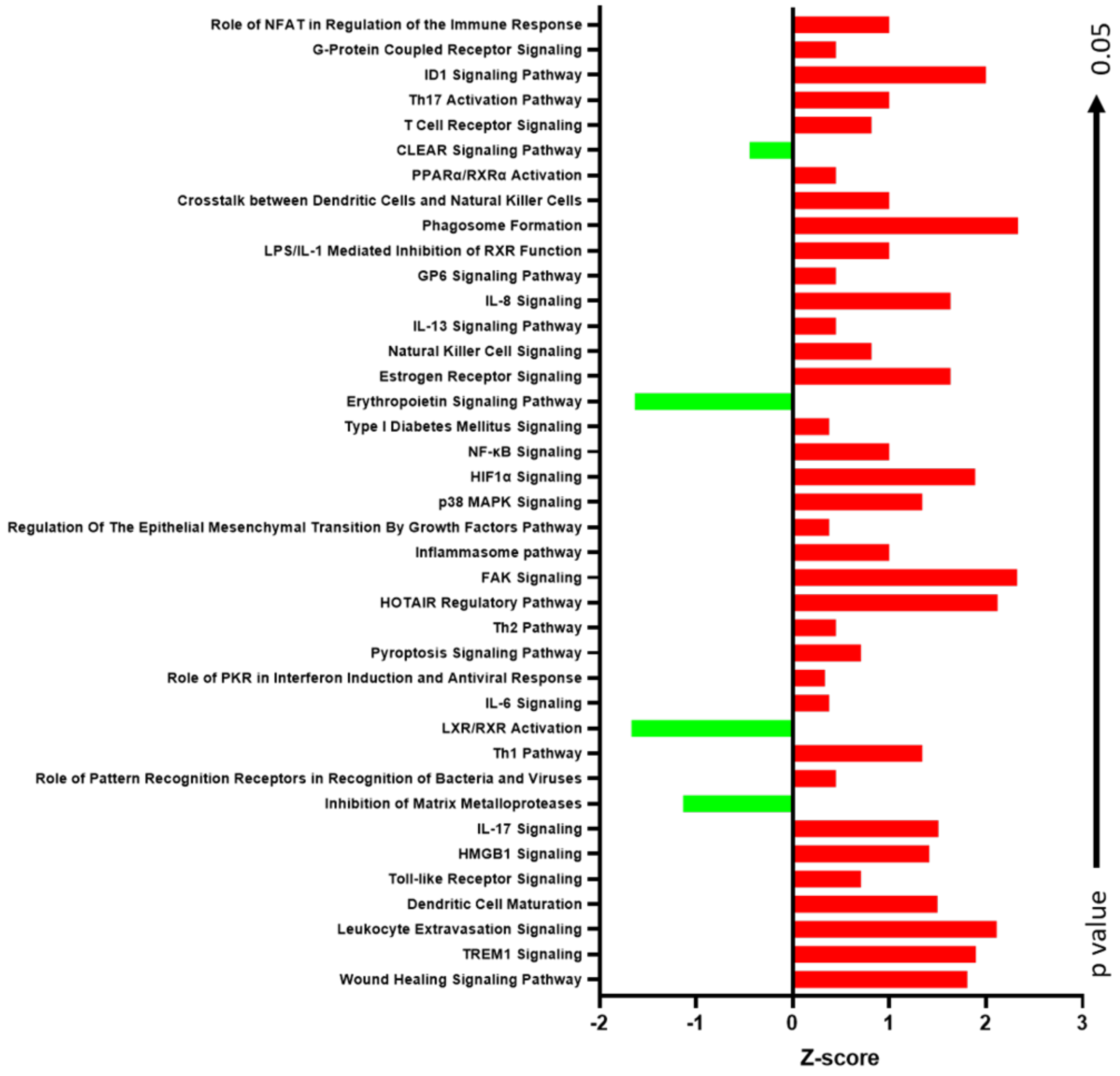

Supplemental Figure S2. Ingenuity Pathway Analysis (IPA) revealed pathways in which mRNA identified to be differentially expressed in tissues surrounding non-releasing sensors 14-d post implantation are highly represented. Native tissue and tissue surrounding control non-releasing sensors were collected at the chronic (14-d) stage of the foreign body response (FBR). Expression of immune gene mRNAs were probed using a custom porcine nanoString panel (254 immune related genes). Ingenuity Pathways Analysis (IPA) was employed to detect and visualize the significant changes in canonical immune signaling genes based on the observed gene expression profiles (Figure 3B). Data are presented as the Bayesian z-scores with associated p-value significance. Z-scores represent the degree of perturbation of the group of pathway genes versus the controls. Positive z-scores represent activation of the pathway (pathway score is dominated by more up-regulated genes), while the negative z-score represents pathway repression (pathway score is dominated by down-regulated genes).

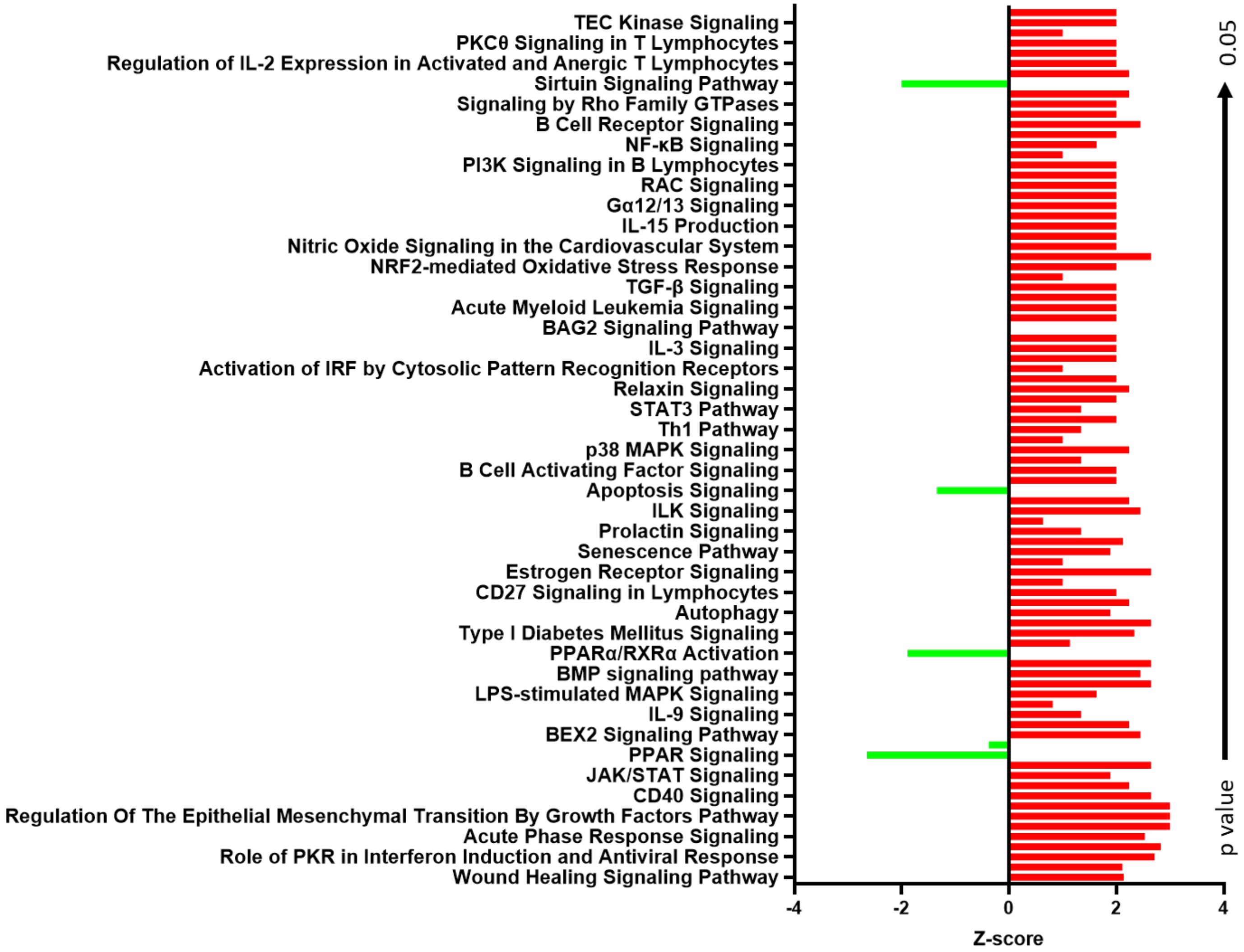

Supplemental Figure S3. Ingenuity Pathway Analysis (IPA) revealed pathways which are differentially expressed in tissues surrounding non-releasing sensors acutely (7-d) and chronically (14-d) post implantation in hyperglycemic animals. Native tissue and tissue surrounding control non-releasing sensors were collected at the acute and (7-d) and chronic (14-d) stages of the foreign body response (FBR) in diabetic pigs. Expression of immune gene mRNAs were probed using a custom porcine nanoString panel (254 immune related genes). Ingenuity Pathways Analysis (IPA) was employed to detect and visualize the significant changes in canonical immune signaling genes based on the observed gene expression profiles. Data are presented as the Bayesian z-scores with associated p-value significance, for **A)** 7-d, and **B)** 14-d post-implantation non-releasing sensors *versus* native tissues. Z-scores represent the degree of perturbation of the group of pathway genes versus the controls. Positive z-scores represent activation of the pathway (pathway score is dominated by more up-regulated genes), while the negative z-score represents pathway repression (pathway score is dominated by down-regulated genes).

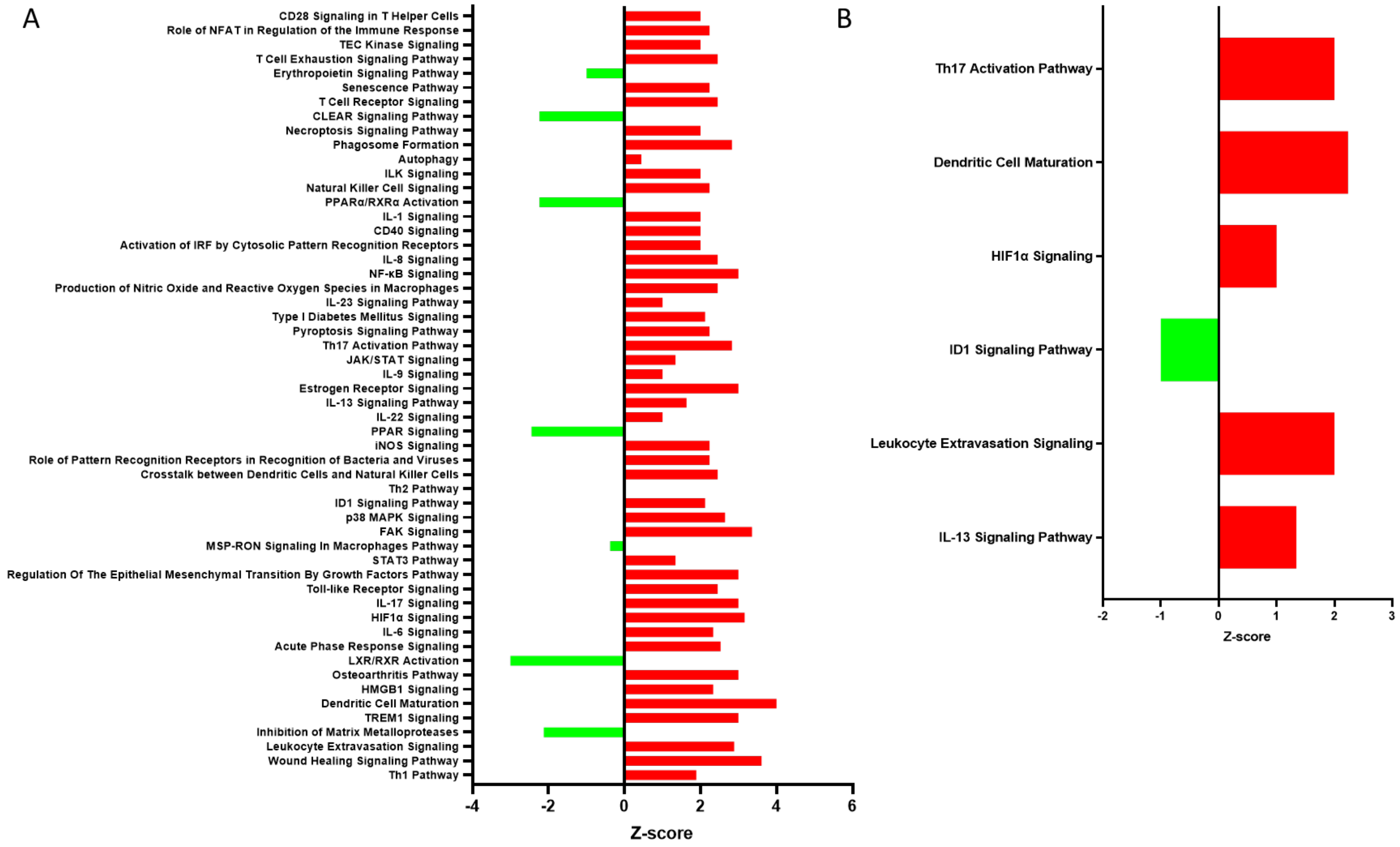

Supplemental Figure S4. Ingenuity Pathway Analysis (IPA) revealed pathways which are differentially expressed in tissues surrounding NO-releasing sensors acutely (7-d) and chronically (14-d) post implantation in hyperglycemic animals. Tissue surrounding NO-releasing or control non-releasing sensors were collected at the acute and (7-d) and chronic (14-d) stages of the foreign body response (FBR) in diabetic pigs. Expression of immune gene mRNAs were probed using a custom porcine nanoString panel (254 immune related genes). Ingenuity Pathways Analysis (IPA) was employed to detect and visualize the significant changes in canonical immune signaling genes based on the observed gene expression profiles. Data are presented as the Bayesian z-scores with associated p-value significance, for **A)** 7-d, and **B)** 14-d post-implantation for NO-releasing sensors *versus* non-releasing sensors. Z-scores represent the degree of perturbation of the group of pathway genes versus the controls. Positive z-scores represent activation of the pathway (pathway score is dominated by more up-regulated genes), while the negative z-score represents pathway repression (pathway score is dominated by down-regulated genes).

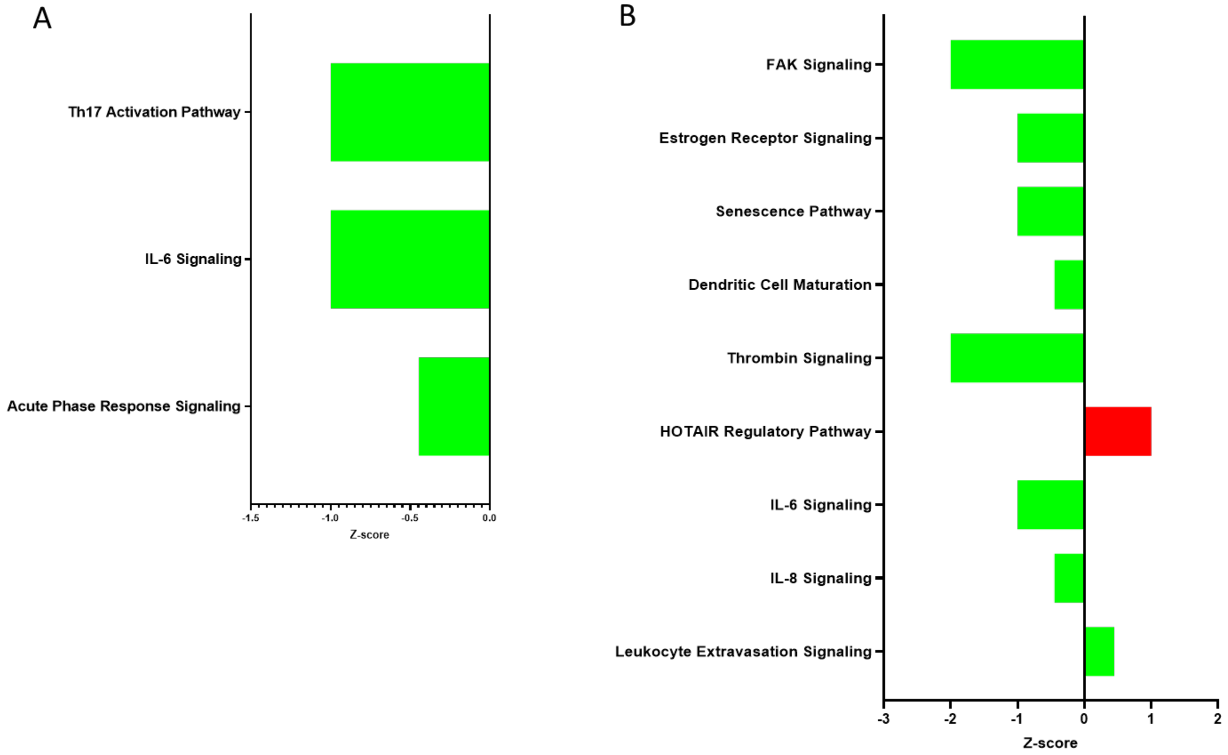

Supplement: Supplementary file 1 [file ijms-23-11635-s001.zip › ijms-1894608-supplementary.pdf]
